# A novel model based on necroptosis to assess progression for polycystic ovary syndrome and identification of potential therapeutic drugs

**DOI:** 10.3389/fendo.2023.1193992

**Published:** 2023-09-07

**Authors:** Mingming Wang, Ke An, Jing Huang, Richard Mprah, Huanhuan Ding

**Affiliations:** ^1^ Department of Physiology, Xuzhou Medical University, Xuzhou, Jiangsu, China; ^2^ Department of Medical Informatics Engineering, Xuzhou Medical University, Xuzhou, Jiangsu, China

**Keywords:** polycystic ovary syndrome, diagnostic model, therapeutic drugs, necroptosis, gene signature

## Abstract

**Background:**

Polycystic ovary syndrome (PCOS), a common endocrine and reproductive disorder, lacks precise diagnostic strategies. Necroptosis was found to be crucial in reproductive and endocrine disorders, but its function in PCOS remains unclear. We aimed to identify differentially diagnostic genes for necroptosis (NDDGs), construct a diagnostic model to assess the progression of PCOS and explore the potential therapeutic drugs.

**Methods:**

Gene expression datasets were combined with weighted gene co-expression network analysis (WGCNA) and necroptosis gene sets to screen the differentially expressed genes for PCOS. Least absolute shrinkage and selection operator (LASSO) regression analysis was used to construct a necroptosis-related gene signatures. Independent risk analyses were performed using nomograms. Pathway enrichment of NDDGs was conducted with the GeneMANIA database and gene set enrichment analysis (GSEA). Immune microenvironment analysis was estimated based on ssGSEA algorithm analysis. The Comparative Toxicogenomics Database (CTD) was used to explore potential therapeutic drugs for NDDGs. The expression of NDDGs was validated in GSE84958, mouse model and clinical samples.

**Results:**

Four necroptosis-related signature genes, IL33, TNFSF10, BCL2 and PYGM, were identified to define necroptosis for PCOS. The areas under curve (AUC) of receiver operating characteristic curve (ROC) for training set and validation in diagnostic risk model were 0.940 and 0.788, respectively. Enrichment analysis showed that NDDGs were enriched in immune-related signaling pathways such as B cells, T cells, and natural killer cells. Immune microenvironment analysis revealed that NDDGs were significantly correlated with 13 markedly different immune cells. A nomogram was constructed based on features that would benefit patients clinically. Several compounds, such as resveratrol, tretinoin, quercetin, curcumin, etc., were mined as therapeutic drugs for PCOS. The expression of the NDDGs in the validated set, animal model and clinical samples was consistent with the results of the training sets.

**Conclusion:**

In this study, 4 NDDGs were identified to be highly effective in assessing the progression and prognosis of PCOS and exploring potential targets for PCOS treatment.

## 1 Introduction

Infertility affects 8-12% of couples of reproductive age and has become a global problem, among which female infertility accounts for 60-70% (1). Polycystic ovary syndrome (PCOS), a common endocrine reproductive disorder, affects 5-20% of women of reproductive age. It is characterized by various reproductive, endocrine, and metabolic features such as oligoovulation, infertility, hyperandrogenemia, obesity, hyperinsulinemia, type 2 diabetes mellitus and cardiovascular diseases (2).

As a highly heterogeneous metabolic syndrome, PCOS is affected by multiple factors, such as race and living environment, and the clinical phenotype varies (3). Recently, Qiao et al. reported the important role of gut microorganisms in the development and treatment of PCOS (4). Another study explored the classification and etiology of PCOS from a genomic perspective (5). Moreover, some groups attempted to investigate the PCOS bio-markers and pathogenic mechanisms using multi-omics and bioinformatics (6–8). Despite the efforts made in recent decades, the etiology of PCOS is not yet fully understood due to its complex pathogenesis and variability. Hence, the diagnosis of PCOS is principally based on the classical Rotterdam criteria and clinical symptoms (9). There is still a lack of precise diagnostic criteria, and many patients often suffer from misdiagnoses or missed diagnoses (10). Given this, it is essential to discover new biomarkers to facilitate timely diagnosis and intervention for PCOS.

Studies have shown that apoptosis of ovarian granulosa cells (GCs) could lead to oocyte apoptosis and follicular atresia, which may be one of the significant factors contributing to the development of PCOS (11–13). Oxidative stress is one of the major causes of GCs apoptosis and ovarian atresia. This process generates sustained high levels of reactive oxygen species, triggering an inflammatory response (14). Inflammation can induce necroptosis in GCs, which may be responsible for the development of PCOS (15).

Necroptosis, a new type of cell death, has recently been considered a potential factor for GC death in the preovulatory follicle (16). Necroptosis, also known as programmed necrosis, is a regulated necrotic cell death mediated by receptor-interacting protein kinase (RIPK) 1 and RIPK3. Features of necroptosis include early loss of cytoplasmic membrane integrity, leakage of cell contents, and swelling of organelles (17). RIPK1 and RIPK3 act as stress sensors to promote necroptosis of GCs (18, 19). It has been shown that the reticulophagy receptor CCPG1 mediated STAT1/STAT3-(p) RIPK1-(p) RIPK3-(p) MLKL pathway could trigger the necroptosis of GCs and be involved in the development of PCOS (12, 20). Moreover, high levels of ROS and immunoinflammatory factors could increase the expression of RIPK1 and RIPK3 in GCs (15). Furthermore, dysregulation of GCs and immune cells in PCOS patients also accelerates anovulation (21). Based on these findings, necroptosis may be a helpful diagnostic tool and therapeutic target for PCOS. However, the gene signatures associated with necroptosis and its mechanism in PCOS remain unclear.

In recent years, the combination of big data analytics and clinical data has become an effective approach for identifying diagnostic markers, exploring pathogenesis, and developing drugs (22). In this study, we utilized machine learning combined with clinical samples to identify necroptosis-related genes, constructed a diagnostic model to assess the progression of PCOS and explored the potential therapeutic drugs for PCOS. This work provides a theoretical basis for identifying new diagnostic markers and therapeutic targets for PCOS.

## 2 Materials and methods

### 2.3 Gene expression profile acquisition and differentially expressed genes screening

We searched the GEO database using the keywords “PCOS, Homo sapiens” and obtained 5 expression datasets: GSE95728, GSE114419, GSE106724, GSE137684, and GSE84958 (23). GSE95728, GSE114419, GSE106724, and GSE137684 were used as the training set and GSE84958 as the validation set. As the training set was from different batches and platforms, it was merged using the sva package version 3.36.0 in R version 3.6.1 to remove batch effects (24). The validation set was normalized with the voom function provided by the limma package (https://bioconductor.org/packages/release/bioc/html/limma.html) version 3.34.7 for model validation analysis (25). Details of the dataset are shown in **Table 1**.

**Table 1:** Gene expression profile information.

Dataset	Database	Sample information	Data source
GSE95728	GEO	7 controls and 7 PCOS patients	GPL16956
GSE114419	GEO	3 controls and 3 PCOS patients	GPL17586
GSE106724	GEO	4 controls and 8 PCOS patients	GPL21096
GSE137684	GEO	4 controls and 8 PCOS patients	GPL16956
GSE84958	GEO	23 controls and 15 PCOS patients	GPL16791
Necroptosis related genes	Published article	159 Necroptosis-related genes	

The samples were divided into control and PCOS groups based on their source information and screened for differentially expressed genes using the limma package in R (version 3.6.1). False discovery rate (FDR)< 0.05 and |log_2_FC|>0.5 were used as the threshold for differential screening.

### 2.2 WGCNA screening for PCOS-related modules and genes

Weighted Gene Co-Expression Network Analysis (WGCNA) could identify modules with similar expression patterns, analyze the linkage between the modules and the phenotype of the samples, map the regulatory network between the modules and identify key regulatory genes. To identify synergistic variation in gene sets, a modular clustering analysis of all genes was performed using the R package WGCNA (Version 1.61, https://cran.rproject.org/web/packages/WGCNA/) (26). In the WGCNA algorithm, the elements of the gene co-expression matrix were defined as the weighted values of the correlation coefficients of the genes. The weights were chosen so that the connections between the genes in each gene network follow a scale-free network distribution. The weighted value here was the softPower. Firstly, by setting a series of powers and calculating the squared correlation coefficients of the connectivity k and p (k) and the average connectivity for each power value, selecting the appropriate power value to make the connections between genes in the network obey the scale-free networks distribution. Secondly, based on the clustering and dynamic pruning method, the parameters (minModuleSize=50: each module contained at least 50 genes; MEDissThres=0.3: modules with similarity > 0.7 will be merged) were set to aggregate highly correlated genes into modules. Finally, calculating the module-phenotype correlation, where the phenotype referred to the disease status of the sample, and the modules closely associated with PCOS were selected as disease-associated genes.

### 2.3 Acquisition of differentially expressed genes for necroptosis

We obtained 159 necroptosis genes from published literature and intersected them with differentially expressed genes and PCOS modular genes to obtain differentially expressed genes for necroptosis (27). The Pearson correlation coefficients and significance between the differentially expressed genes were calculated based on the correspondence of the PCOS samples.

### 2.4 Functional enrichment analysis

Gene Ontology (GO) was used to analyze the biological function, pathway or cellular localization of differentially enriched genes. Kyoto Encyclopedia of Genes and Genomes (KEGG) was a functional enrichment database for identifying the pathways in which a gene set (multiple genes) might be significantly concentrated. Gene-Set Enrichment Analysis (GSEA) could determine whether gene sets differed significantly between two biological states. GO and KEGG enrichment analysis was performed with DAVID 6.8. Enrichment results were considered significant for each enriched item containing at least 2 genes and p<0.05. The top 20 items were selected for presentation (28–30). Moreover, the “cluster Profiler” package (Version 1.2.1) was used to conduct GSEA. Significant p-values were obtained using the “BH” correction, with p.adjust<0.05 considered a significant enrichment (31).

### 2.5 Protein-protein interaction network construction with GeneMANIA database

The GeneMANIA database (http://genemania.org/) was used to perform a PPI analysis of model genes and their 20 interacting genes to predict correlations between co-localization, shared protein structural domains, co-expression and pathways (32).

### 2.6 Diagnostic model construction

The Least Absolute Shrinkage and Selection Operator (LASSO) logistic regression model was used to further screen for PCOS-associated necroptosis genes with the glmnet package (version 2.0-18) in R3.6.1 language (33). The Riskscore model was constructed based on the gene regression coefficients and expression levels. The Riskscore was calculated as follows:


$$\text{Riskscore}=\sum {\text{β}}_{\text{gene}}\times {\text{Exp}}_{\text{gene}}$$

β_gene_ represents the LASSO regression coefficient of the gene and Exp_gene_ represents the expression level of the gene in each sample. To validate the model accuracy, the riskscore values for each sample in the validation dataset were calculated to plot the diagnostic ROC curves, box plots of the distribution of RiskScore and heat maps of the expression of the model genes, following the Riskscore formula and using the same regression coefficients.

### 2.7 The construction and visualization of nomogram

Nomogram was established based on multifactor regression analysis, which integrated multiple predictors and plotted them on the same plane at a certain scale to show the interrelationships of variables in the predictive model. Nomogram was constructed using the rms package Version 5.1-2 in the R (34). Calibration curves were used to assess the predictive power, decision curve analysis (DCA) to assess the clinical utility and the Concordance index (C-index) to evaluate the predictive power of the nomogram.

### 2.8 Immune microenvironment analysis

The ssGSEA algorithm was used to calculate individual immune cell enrichment scores using the R package GSVA (version 1.36.2). Using the Wilcoxon test and Spearman analysis, immune cells with a p< 0.01 were considered significantly correlated with PCOS (35).

### 2.9 Drugs prediction

To explore chemical drugs associated with diagnostic genes, we searched for the targeting drugs (Chemical Interactions) of the key genes as described above in the online CTD database (https://ctdbase.org/) (36). Drug-gene relationship pairs were selected to be supported by at least two references and PPI network construction was performed using Cytoscape software (version 3.4.0, http://chianti.ucsd.edu/cytoscape-3.4.0/) (37).

### 2.10 PCOS patients’ samples collection and mouse model construction

The details of GCs collection from PCOS patients and controls are described in a previous publication (13). Briefly, follicular fluid was centrifuged at 250 x g for 10 minutes after removing the oocytes. The GCs layer was pipetted into a new centrifuge tube, washed, resuspended in PBS, centrifuged at 250 x g for 5 minutes and the cell was collected.

The PCOS mouse model construction details were described in a previous publication (13). Briefly, 3-week-old female mice were injected subcutaneously with DHEA (6 mg/100 g body weight) daily and controls were injected with an equivalent dose of sesame oil. Twenty-eight (28) days later, blood was collected from the eyes and ovarian tissue was collected for testing and analysis (28).

### 2.11 Gene expression validation with *real-time quantitative PCR*

Gene expression with qPCR has been previously described (12, 38). Briefly, mouse ovarian tissue and GCs from PCOS patients were extracted using the TRIzol method and reverse transcribed using a cDNA kit. The hub gene expression was measured using qPCR using Glyceraldehyde phosphate dehydrogenase (GAPDH) as an internal control. The mRNA expression levels were determined using the 2^−ΔΔCt^ method. The primer sequences are listed in **Table S1**.

## 3 Results

### 3.1 Acquisition of differentially expressed genes for necroptosis

**Figure 1:**
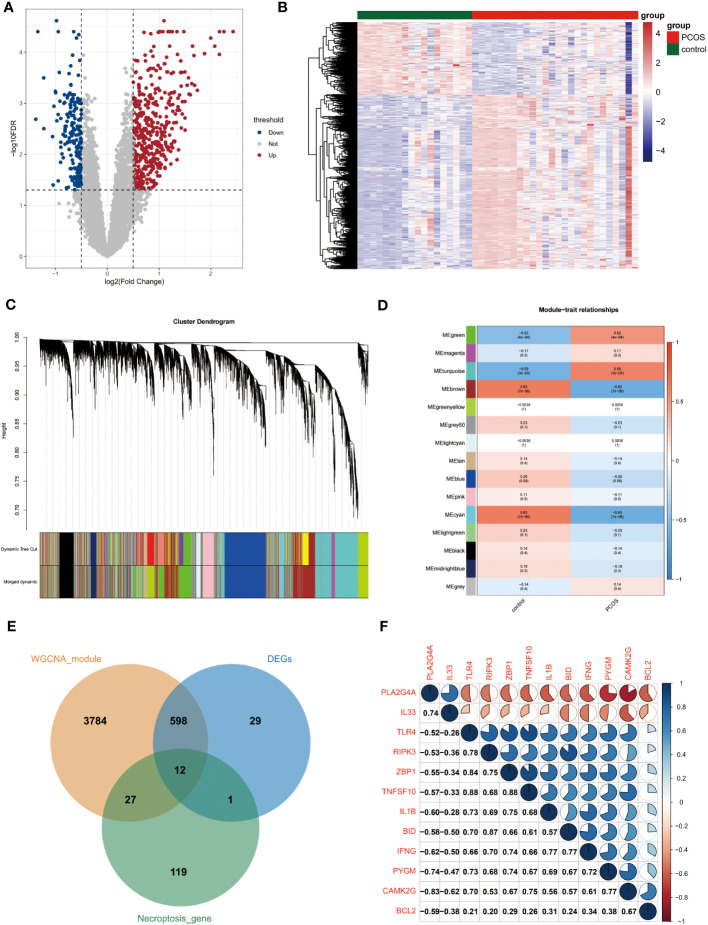
Acquisition of differentially expressed genes for necroptosis. **(A)** Volcano plot of differentially expressed genes. Red and blue dots indicate upregulated and down-regulated genes, respectively, and gray is the non-significant gene. **(B)** Heat map of differential gene expression. Blue is low-expressed genes and red is high-expressed genes. **(C)** Systematic clustering tree of genes and gene modules generated by dynamic shearing method. Different colors represent different gene modules. **(D)** Correlation analysis of WGCNA modules with the disease status of the samples. Numbers outside parentheses indicate correlation coefficients and those inside the parentheses indicate p-values. **(E)** The intersection of necroptosis genes with differentially expressed genes and PCOS module genes. **(F)** Correlation analysis of PCOS-associated differential necroptosis genes. Red sectors indicate negative correlations, while blue sectors indicate positive correlations. The larger fan area indicates the larger absolute value of the correlation coefficient. Numbers in the lower left corner indicate specific correlation coefficients. WGCNA, weighted gene co-expression network analysis.

The four sets of gene expression profiles were combined into one dataset by removing the batch effect (**Supplementary Figure 1**, **Figure S1**). A total of 453 upregulated and 187 down-regulated genes were obtained (**Figures 1A, B**).

We performed WGCNA analysis using the full gene expression matrix to screen for genes associated with PCOS. Based on clustering and dynamic pruning methods, highly correlated genes were clustered and eventually integrated into 14 modules (**Figures S2**, **1C**). As shown in **Figure 1D**, the negative correlation coefficients between the brown and cyan modules and PCOS are significant. In contrast, the positive correlation coefficients between the green and turquoise modules and PCOS are remarkable, so the four module genes (4421 genes in total) were considered closely related module genes for PCOS (**Figure 1D**).

A total of 12 differentially expressed genes for necroptosis were obtained by taking the intersection of necroptosis genes with differentially expressed genes and PCOS module genes (**Figure 1E**). The correlation coefficients between two of the 12 genes were further calculated and heat maps were created (**Figure 1F**).

### 3.2 Functional enrichment analysis

**Figure 2:**
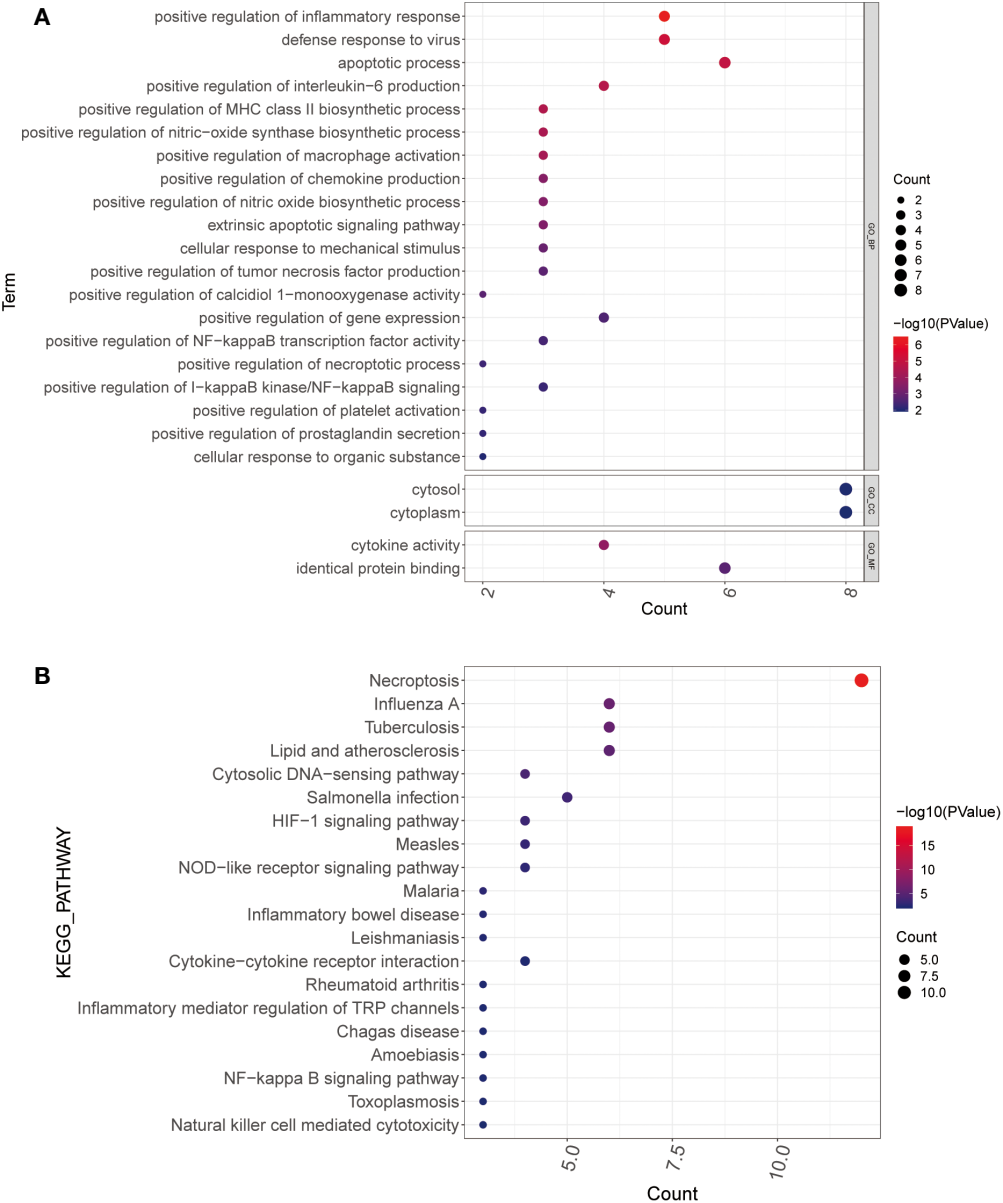
Functional enrichment analysis. **(A, B)** GO **(A)** and KEGG **(B)** analysis of PCOS-associated differential necroptosis genes. The color blue to red indicates the significance from less to more, and the bubble size indicates the number of enriched genes GO, gene ontology; KEGG, Kyoto encyclopedia of genes and genomes.

GO and KEGG enrichment analyses were performed to determine the functions and related pathways of the differentially expressed genes for necroptosis. We found 102 GO items and 31 KEGG pathways were enriched. The GO analysis showed enrichment of positive regulation of inflammatory response, defense response to virus, apoptotic process, regulation of interleukin-6 production, regulation of MHC class II biosynthetic process, positive regulation of tumor necrosis factor production, and positive regulation of necroptotic process in biological process. Moreover, we observed an enrichment of cell membranes, cytoplasm in cellular components. Furthermore, cytokine activity and identical protein binding were enriched in molecular functions (**Figure 2A**). Most importantly, the KEGG mainly focused on necroptosis, HIF-1 signaling pathway, NOD-like receptor signaling pathway, inflammatory mediator regulation of TRP channels, NF-kappa B signaling pathway, and Natural killer cell-mediated cytotoxicity (**Figure 2B**).

### 3.3 LASSO algorithm to screen for differentially diagnostic genes for necroptosis (NDDGs)

**Figure 3:**
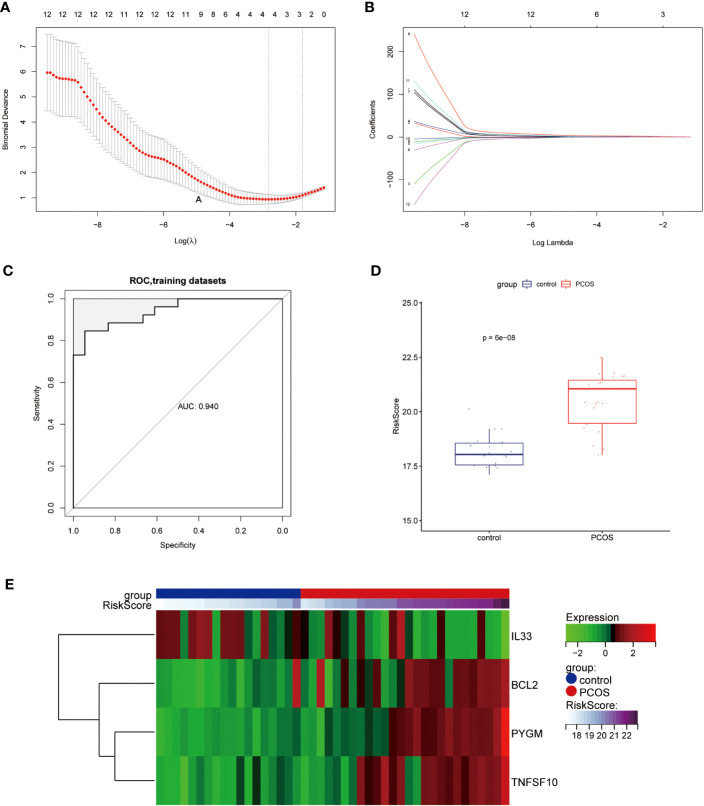
Construction of the diagnostic scoring model **(A, B)** LASSO logistic regression algorithm used to screen key genes. **(C)** ROC curves of the diagnostic model in the training set. **(D)** Distribution of RiskScore in control and PCOS groups. **(E)** Heat map of the expression of the NDDGs in each sample. LASSO, least absolute shrinkage and selection operator; ROC, receiver operating characteristic curve; NDDGs, differentially diagnostic genes for necroptosis.

Applying the LASSO Cox regression algorithm, we obtained four NDDGs BCL2, IL33, PYGM, and TNFSF10 in the training set, which was used as a necroptosis signature (**Figures 3A, B**). Furthermore, we constructed RiskScore model with the corresponding regression coefficients of NDDGs. The receiver operating characteristic curve (ROC) showed that the area under the curve (AUC) was 0.94, indicating an excellent disease prediction effect (**Figure 3C**). Moreover, the RiskScore was significantly higher in PCOS group than in control, which evidenced the accuracy of the model (**Figure 3D**). Furthermore, we have illustrated the relationship between the expression of the NDDGs and the RiskScore by using a heat map (**Figure 3E**). More importantly, we re-confirmed this model’s accuracy with the same method in the validation set (**Figure S3**). All the results proved that the model has a good prediction effect.

### 3.4 The construction of a nomogramand ROC curve

**Figure 4:**
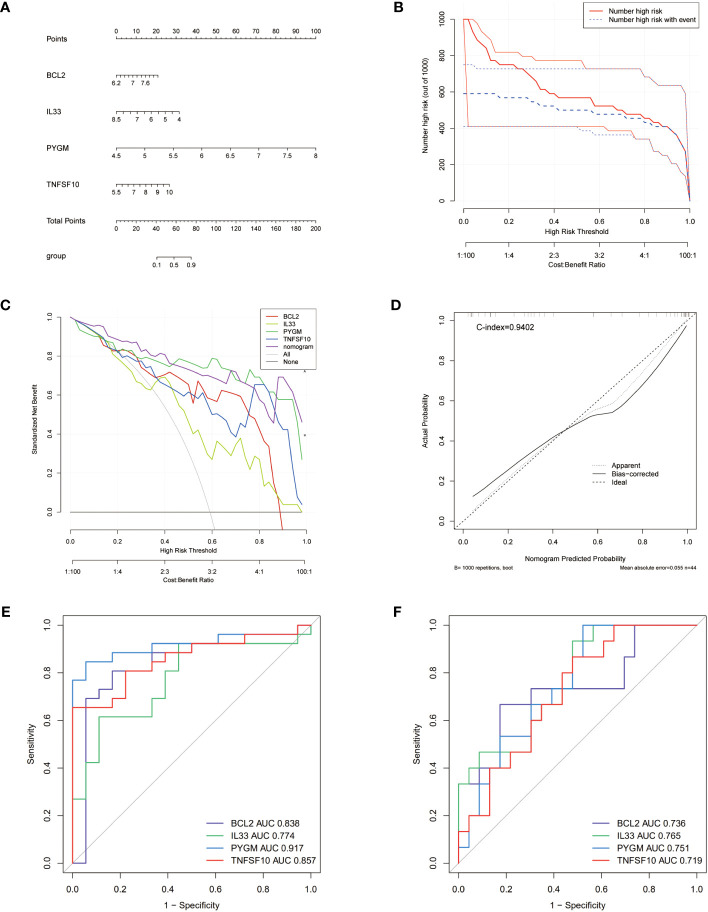
Nomogram for PCOS patients and ROC curves. **(A)** Nomogram for PCOS patients. **(B)** Calibration curve to assess the predictive power of the nomogram. **(C)** DCA curve to assess the clinical value of the nomogram. **(D)** Clinical effects curve based on DCA curves to assess the clinical impact of the nomogram. DCA, decision curve analysis. **(E, F)** ROC curves analysis of training set **(E)**, testing set **(F)**.

To validate the diagnostic ability of the 4 NDDGs for PCOS, we constructed a nomogram and used calibration curves to assess its predictability (**Figure 4A**). The calibration curve showed a minor error with a C-index of 0.878 between the actual PCOS risk and the predicted risk, indicating that the nomogram has a high PCOS predictive accuracy (**Figure 4B**). Moreover, the decision curve analysis (DCA) showed a favorable clinical benefit for the nomogram of patients (**Figure 4C**). To better assess the clinical effect of the nomogram, a clinical impact curve was plotted based on the DCA curve. As shown in **Figure 4D**, the “Number high risk” curve is in proximity to the “Number high risk with event” curve for high-risk thresholds from 0.7 to 1, suggesting that the nomogram has a desirable performance in prediction.

To further evaluate the prognosis of NDDGs in PCOS patients, we conducted ROC curve analysis in both the training and validation cohorts. The results showed that NDDGs have high accuracy in predicting patient outcomes in both two cohorts. (**Figures 4E, F**)

### 3.5 PPI construction and GSEA enrichment analysis of NDDGs

**Figure 5:**
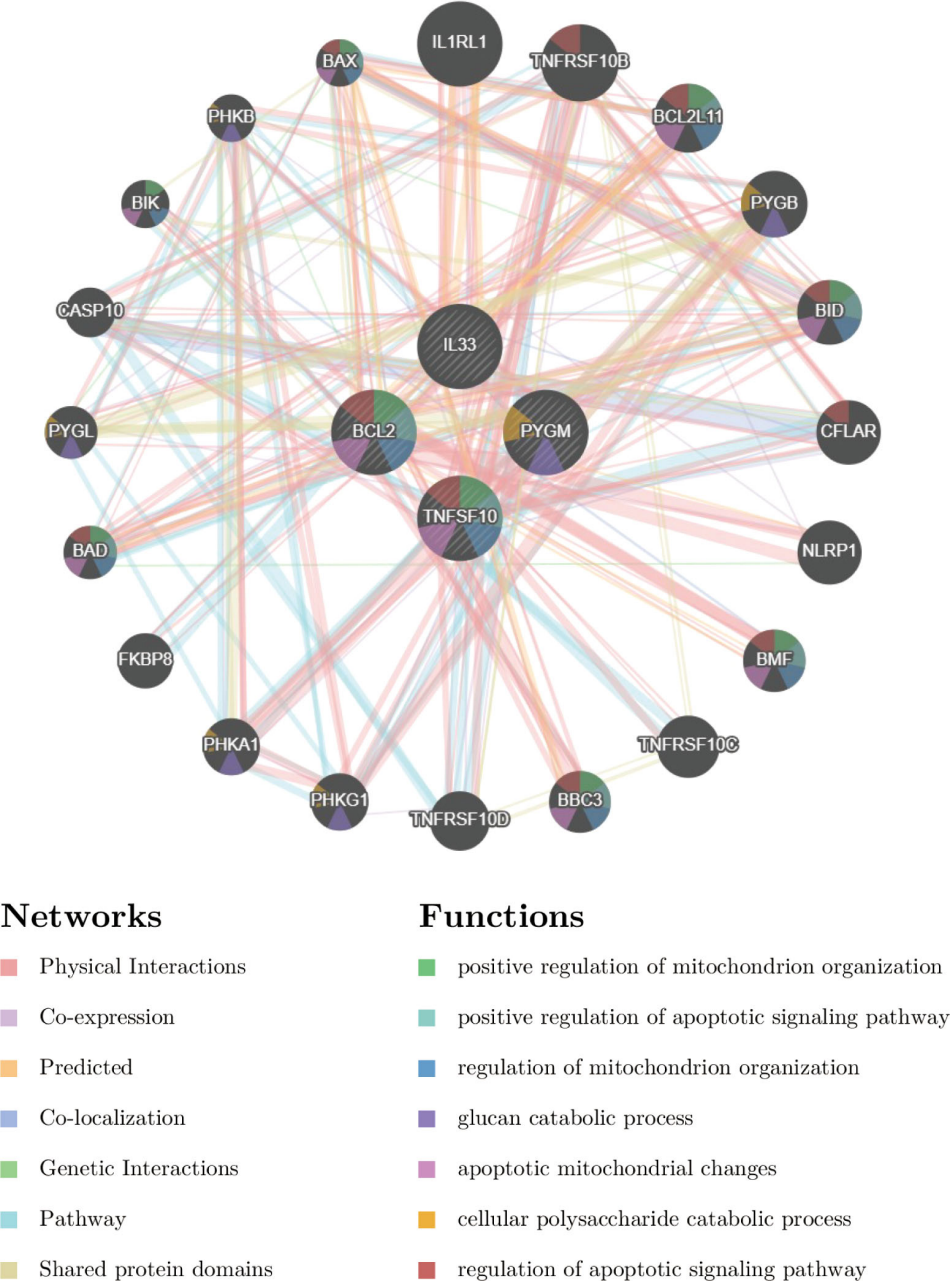
Construction of PPI network with GeneMANIA database.

We constructed a PPI network and performed functional analysis for the 4 NDDGs with their 20 reciprocal genes using the GeneMANIA database. As shown in **Figure 5**, NDDGs were involved in apoptotic signaling pathways and mitochondria-related functional pathways.

**Figure 6:**
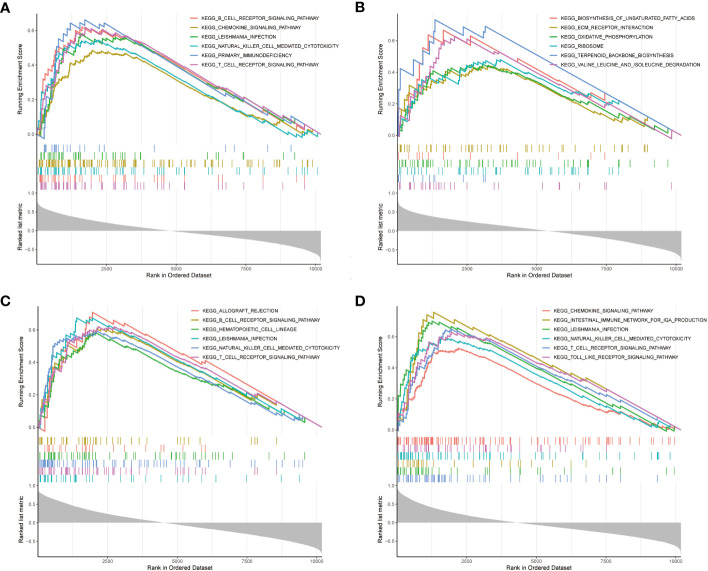
GSEA enrichment analysis **(A–D)** GSEA enrichment analysis of NDDGs of BCL2 **(A)**, IL33 **(B)**, PYGM **(C)** and TNFSF10 **(D)**. GSEA, gene set enrichment analysis.

Furthermore, GSEA enrichment analysis was used to mine the mechanism of KEGG pathway for NDDGs **Figure 6**. The present results demonstrate the positively correlated top 6 KEGG pathways for each gene. The findings show that the pathways were significantly enriched to T cells as natural killer cell-related pathways, which implied the crucial roles of the immune microenvironment in PCOS.

### 3.6 Relationships between the NDDGs and the immune microenvironment

**Figure 7:**
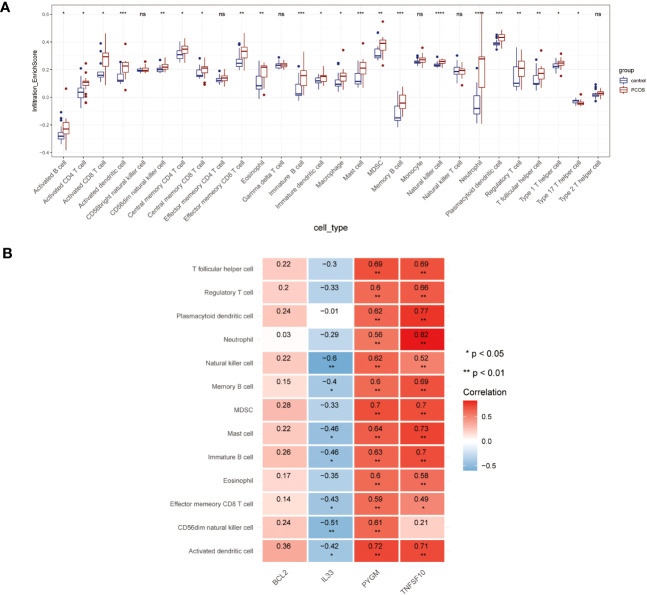
Immune microenvironment analysis. **(A)** The distribution of 28 immune cells in PCOS and control group. **(B)** Correlation analysis of 4 diagnostic necroptosis genes with 13 immune cell species. (* p< 0.05, ** p< 0.01, *** p< 0.001, **** p< 0.0001, ns indicates not significant).

In the GSEA analyses, we found that NDDGs were highly enriched in immune-related pathways. We further employed ssGSEA algorithm to evaluate the association between necroptosis and the immune microenvironment in PCOS (**Figure 7A**). Among 28 immune cells, we identified 13 immune cells that differed significantly between control and PCOS and were noticeably enriched in the PCOS group.

Furthermore, we estimated the correlation efficiency between NDDGs and the immune microenvironment. Among them, PYGM and TNFSF10 were predominantly favorably correlated with the immune cells while IL33 was primarily negatively correlated with natural killer cell, memory B cell, mast cell, immature B cell, effector memory CD8 T cell, CD56dim natural killer cell, activated dendritic cell (**Figure 7B**).

### 3.7 Validation of NDDGs and drug prediction

**Figure 8:**
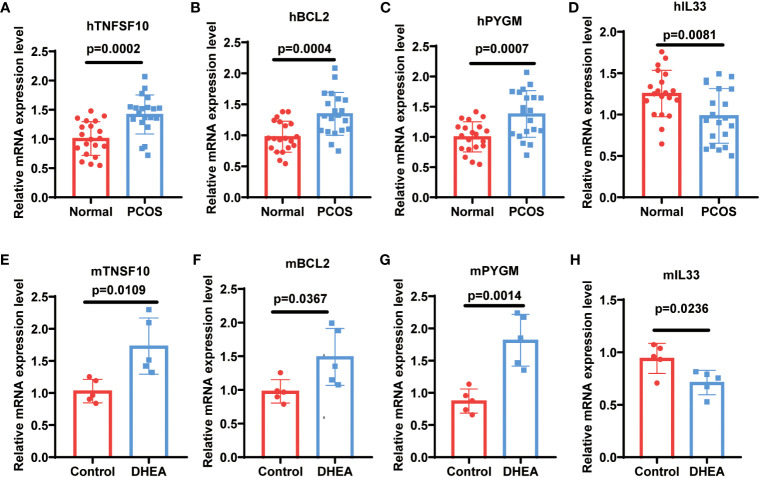
Validation of NDDGs in PCOS patients and mouse model. **(A–D)** The expression level of TNFSF10, BCL2, PYGM and IL33 in granulosa cells from PCOS patients and controls. **(E–H)** The expression level of TNFSF10, BCL2, PYGM and IL33 in the ovarian tissues of PCOS mice and controls.

To validate the expression of NDDGs in PCOS, we first tested the expression of NDDGs using the validation set GSE84958, which showed significant differences in BCL2 (Fold change (FC) = 1.5), PYGM (FC = 1.87) and IL33 (FC = 1.76) (**Figure S4**). More importantly, we collected clinical samples and constructed a PCOS mouse model for further validation. The results exhibited that the expression of BCL2 (FC = 1.35 & 1.52), PYGM (FC = 1.38 & 2.08) and TNFSF10 (FC = 1.42 & 1.73) was significantly higher in the GCs from PCOS patients and ovarian tissues of PCOS mice compared with controls. In comparison, the expression of IL33 (FC = 1.27 & 1.32) was notably lower (**Figure 8**). In general, these results were consistent with the analysis of the training set.

**Figure 9:**
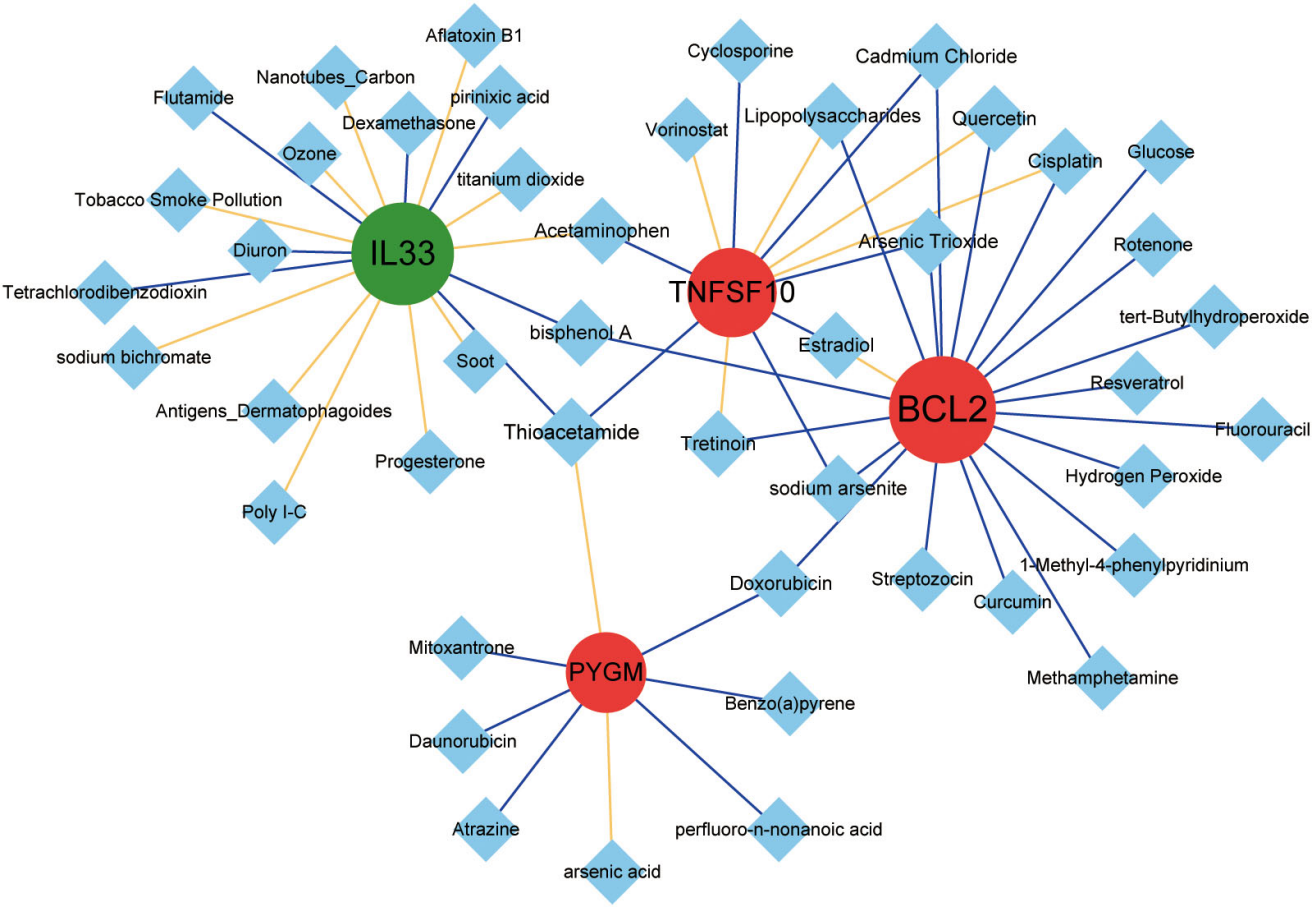
Drug prediction for the NDDGs. Red circles indicate upregulated genes, green circles indicate downregulated genes, and blue diamonds indicate drug small molecules. The yellow line represents a drug that increases the expression of a gene or protein, and the blue line represents a drug that decreases the expression of a gene or protein. The node size denotes the connectivity size.

To predict the corresponding drugs of NDDGs, we searched the CTD and constructed a network diagram. We discovered 58 drug-gene relationship pairs in the database containing 4 NDDGs and 45 drug molecules (**Figure 9**).

## 4 Discussion

PCOS is one of the most common endocrine disorders in women of reproductive age. PCOS patients are often misdiagnosed or underdiagnosed due to the lack of precise diagnostic criteria. Discovering new and effective biomarkers may help facilitate timely diagnosis and intervention for patients with PCOS. In this study, we construct a risk model based on necroptosis to assess the progression of PCOS. Based on machine learning and algorithmic analysis, we identified four necroptosis-related signature genes, IL33, BCL2, PYGM, and TNFSF10, and further determined the signaling pathways, immune microenvironment and targeted drugs.

In PCOS patients, the prevalence of insulin resistance is around 50-70% (39). Ovarian GCs are energy-consuming cells providing estradiol and nutrients to the oocyte (40). Consequently, insulin resistance or apoptosis in GCs can impair oocyte development and ovulation, an essential cause of PCOS (41, 42). PI3K/AKT-mediated insulin signaling pathway was closely associated with metabolic abnormalities and reproductive disorders in PCOS (43). Insulin could activate AS160, GSK-3β and FOXO1 through PI3K/AKT to promote GLUT4 transport and glucose uptake and to regulate gluconeogenesis and glycogen synthesis (44). Insulin resistance impairs glucose metabolism and imposes a hyperfunctional state on the ovaries, which increases the response to gonadotropins, leads to abnormal steroid hormone synthesis and secretion, and results in excessive follicular recruitment and development (44). In PCOS patients, insulin resistance in GCs leads to PI3K/AKT signaling inhibition, contributing to GCs apoptosis and degeneration, subsequently leading to follicular atresia (45). Besides, Insulin resistance is an important cause of hyperglycemia in women with PCOS. Hyperglycemia-derived ROS led to the activation of NF-kB and the production of pro-inflammatory factors such as TNF or IL-6 (46, 47). High glucose caused the inhibition of antioxidant enzymes, reducing the ability of GCs to remove ROS (48). The accumulation of ROS impairs the function of GCs and causes GCs apoptosis and follicular atresia. As such, dysregulation of insulin signaling and high glucose production leads to inflammation and GCs apoptosis, which might be responsible for antral follicular atresia and the development of PCOS (49).

PYGM is a muscle glycogen phosphorylase reported to have a primary role in providing energy for muscle contraction. It is also expressed in tissues other than muscle, such as the brain, lymphoid tissue, blood and ovaries (50, 51). PYGM was reportedly involved in insulin and glycogen signaling pathways, insulin resistance and necroptosis (51). Based on the increased expression of PYGM in GCs reported in this study, we speculate that PYGM may influence PCOS development via insulin resistance regulation in GCs. Besides, PYGM was also proven to regulate the immune function of T cells (52). Our immune infiltration results revealed a significant correlation between PYGM and various T cells. Hence, PYGM may influence the inflammatory status of PCOS by regulating the immune infiltration of T cells. However, the mechanisms underlying these biological functions need to be thoroughly investigated.

TNFSF10 belongs to a family of tumor necrosis factor (TNF) ligands that induce apoptosis by binding to their receptors and triggering the activation of MAPK8/JNK, caspase 8 and caspase 3 (53). It was reported that TNFSF10 could be a marker of necroptosis in various diseases, such as ischemic cardiomyopathy and cancers (54, 55). This study is the first to identify TNFSF10 as a diagnostic marker of necroptosis in PCOS, and its specific function in the development of PCOS needs to be further investigated. BCL2 has been reported to be involved in the development of PCOS (56). Also, BCL2 was involved in necroptosis in various diseases (57, 58). In this study, it acted as one of the diagnostic markers of necroptosis in PCOS, consistent with the previous reports (59).

IL33 is a new member of the IL1 cytokine family and encodes a cytokine protein. It can function either extracellularly as an inflammatory factor or intracellularly as a nuclear factor with transcriptional regulatory functions (60). IL33 is mainly involved in the maturation of immune cells such as Th2 cells, the activation of mast cells and natural killer cells. One study found that IL33 expression was elevated in PCOS patients compared to controls (61). Another showed a significant decrease in IL33 levels in the ovaries of PCOS rats treated with omega-6 fatty acids (62). These results implied that IL-33 might be involved in the development of PCOS as a pro-inflammatory factor. However, the decreased expression of IL-33 in GCs from PCOS patients found in the public database in the present study was not consistent with the reports. The reason for this may be the method of analysis and the different sources, types, and size of the samples. Instead of using the serum of patients and ovarian tissue from rats, we obtained the results using several databases to analyze PCOS GCs. More importantly, we verified the expression of IL33 in the validation set GSE84958, clinical sample and mouse model. Despite this, the reasons for the discrepancy in results require further analysis.

The GO category “positive regulation of inflammatory response, apoptotic process, positive regulation of interleukin-6 production, positive regulation of macrophage activation, positive regulation of tumor necrosis factor production, positive regulation of necroptotic process, T cell homeostasis” indicated that the genes were involved in processes of immune regulation, which fits well with the concept of PCOS as a chronic inflammatory disease. KEGG enrichment analysis was significantly linked to necroptosis. The enriched GSEA pathways involve inflammatory, immune, and apoptotic signaling pathways. These pathways have included the T cell receptor signaling pathway, natural killer cell-mediated cytotoxicity, toll-like receptor signaling pathway, B cell receptor signaling pathway, and cytokine receptor interaction, critical in the inflammatory response to PCOS.

By analyzing the correlation between diagnostic genes and immune cells, we found that PYMG and TNFSF10 were positively correlated with immature B cells, memory B cells, effector memory CD8 T cell, regulatory T cell, T follicular helper cell and IL33 showed a negative correlation with immature B cell, memory B cells. Both T helper cell 17 (T17) and Treg cells belong to CD4+T lymphocytes. Chronic low-grade inflammation was considered a key factor in the pathogenesis of PCOS (63). The imbalance of T cell subsets and the abnormal cytokine concentrations exist in the ovary of women with PCOS (64). PYGM is also expressed in T lymphocytes, where it plays a crucial role in the control of IL2–stimulated T-cell migration and proliferation in an EGFR-dependent manner (52, 65). EGF/EGFR signaling was reported to affect the proliferation of cumulus GCs, oocyte maturation and meiosis and play a potential role in the pathogenesis of PCOS (66). We hypothesize that EGFR may activate PYGM to regulate T cell migration and proliferation in PCOS cases. TNFSF10, also known as TRAIL, regulates immune responses and cell homeostasis via an apoptosis-independent pathway (67). TRAIL/TRAIL-R interaction regulates CD4^+^ T cell activation and directly suppresses T cell activation via inhibiting TCR signaling, indicating that TRAIL-R is a novel immune checkpoint in T cell responses (68). In the present study, TNFSF10 was observed to have a strong positive correlation with CD4+ T cells, suggesting that it may contribute to immunomodulatory effects in PCOS by modulating the T-cell response.

As reported, CD19+ B cells could contribute to the pathogenesis of PCOS (69). The peripheral proportion and activity of CD19+ B cells were increased in women with PCOS. DHEA-induced morphological changes to mouse ovaries could be prevented by CD19+ B cell depletion. Moreover, TNF-α-producing B cells are involved in the pathological process of PCOS (70). In this study, GSEA analysis showed that NDDGs were enriched in the B cell receptor signaling pathway. Immune cell analysis indicated immature B cells and memory B cell numbers were significantly higher in PCOS samples. Meanwhile, PYGM, TNFSF10 and IL33 were notably associated with immune infiltration of B cells. It has been found that TNFSF10 was demonstrated to be upregulated in B cells in primary Sjögren’s syndrome, suggesting a regulatory role for TNFSF10 in B cells. Our subsequent concern may be how these genes regulate B-cell function in PCOS.

We predicted compounds corresponding to the four diagnostic genes from the CTD database and discovered several compounds, such as resveratrol, tretinoin, quercetin, curcumin, etc., which have been investigated as therapeutic drugs for PCOS (71–73). For instance, quercetin has been demonstrated to reduce inflammation in PCOS patients and DHEA-induced PCOS rats (74, 75). Another study showed that curcumin might be a safe and useful supplement to ameliorate PCOS-associated hyperandrogenemia, hyperglycemia, and hyperlipidemia (76, 77).

Overall, we found IL33, BCL2, PYMG, and TNFSF10 to be potentially necrotic apoptosis-associated diagnostic markers in PCOS. Nonetheless, this study has some limitations. The results were based on mining and analyzing the published database. There was still a lack of experimental evidence for the mechanism of these diagnostic markers in necroptosis of GCs and the development of PCOS. In particular, whether PYMG is involved in the development of PCOS by affecting insulin resistance and inflammation needs to be thoroughly investigated. Second, the cellular composition of the immune infiltrate was significantly different in PCOS patients compared with normal. Primarily, the activated dendritic cell, immature B cell, mast cell, MDSC, memory B cell, natural killer cell, neutrophil, and plasmacytoid dendritic cell may be associated with GCs necroptosis and the development of PCOS. Further studies of these diagnostic genes and immune cells may provide feasible directions for clinical diagnosis and immunotherapy of PCOS.

## 5 Conclusion

In this study, we constructed a risk model based on necroptosis to assess the progression of PCOS. Based on machine learning and algorithmic analysis, we identified four necroptosis-related signature genes, IL33, BCL2, PYMG, and TNFSF10, and further determined the signaling pathways, immune microenvironment, and targeted drugs. Furthermore, the expression of the four model genes was validated in a PCOS mouse model and clinical samples. This is the first necroptosis-related signature in PCOS and could be a valuable and non-invasive tool for diagnosing and evaluating the progression and prognosis of PCOS patients. This study provides a theoretical basis and new insights into the pathogenic mechanism and therapeutic drug development for PCOS.

## Data availability statement

The datasets presented in this study can be found in online repositories. The names of the repository/repositories and accession number(s) can be found in the article/**Supplementary Material**.

## Ethics statement

The studies involving humans were approved by The Ethics Committee of the Medical University of Bialystok. The studies were conducted in accordance with the local legislation and institutional requirements. The participants provided their written informed consent to participate in this study. The animal study was approved by The Ethics Committee of China Agricultural University. The study was conducted in accordance with the local legislation and institutional requirements.

## Author contributions

MW and KA contributed to the data collection, qPCR analysis and manuscript writing. RM contributed to the manuscript revising. JH contributed to data collection. HD contributed to the data analysis and writing. All authors contributed to the article and approved the submitted version.
