# Corrigendum: A novel model based on necroptosis to assess progression for polycystic ovary syndrome and identification of potential therapeutic drugs

**DOI:** 10.3389/fendo.2024.1432697

**Published:** 2024-05-24

**Authors:** Mingming Wang, Ke An, Jing Huang, Richard Mprah, Huanhuan Ding

**Affiliations:** ^1^Department of Physiology, Xuzhou Medical University, Xuzhou, Jiangsu, China; ^2^Department of Medical Informatics Engineering, Xuzhou Medical University, Xuzhou, Jiangsu, China

**Keywords:** polycystic ovary syndrome, diagnostic model, therapeutic drugs, necroptosis, gene signature

**Incorrect Funding**

In the published article, there was a mistake in the Funding statement. The grant number for the Natural Science Foundation of Jiangsu was displayed as BK2021090. The correct grant number is BK20210904.

The authors apologize for this error and state that this does not change the scientific conclusions of the article in any way. The original article has been updated.

