# Corrigendum: A novel model based on necroptosis to assess progression for polycystic ovary syndrome and identification of potential therapeutic drugs

**DOI:** 10.3389/fendo.2024.1502142

**Published:** 2024-10-24

**Authors:** Mingming Wang, Ke An, Jing Huang, Richard Mprah, Huanhuan Ding

**Affiliations:** ^1^ Department of Physiology, Xuzhou Medical University, Xuzhou, Jiangsu, China; ^2^ Department of Medical Informatics Engineering, Xuzhou Medical University, Xuzhou, Jiangsu, China

**Keywords:** polycystic ovary syndrome, diagnostic model, therapeutic drugs, necroptosis, gene signature

In the published article, there was a mistake in the Funding statement. The grant number for the Jiangsu Higher Education Institutions of China was incorrect (Grant No. KY13022201). The correct grant number is 22KJB180007.

The authors apologize for this error and state that this does not change the scientific conclusions of the article in any way. The original article has been updated.

