# Supplementary material for: A novel model based on necroptosis to assess progression for polycystic ovary syndrome and identification of potential therapeutic drugs
Source: Front Endocrinol (Lausanne). 2023 Sep 7;14:1193992. doi: 10.3389/fendo.2023.1193992 (PMC10517861; doi:10.3389/fendo.2023.1193992)

**Supplementary Figure (Figure S) legends**

**Figure S1 Distributions of data before (A) and after (B) removal of batch effects in GSE95728, GSE114419, GSE106724, and GSE137684**

**Figure S2 WGCNA screening of PCOS-related modules and genes**

**A & B.** WGCNA power values are plotted. **A.** Soft Threshold (power) indicates the weight, and the vertical coordinate indicates the square of the correlation coefficient between the connectivity k and p(k). The higher the square of the correlation coefficient, the more the network approximates the distribution of the scale-free network.

**B.** Soft Threshold (power) denotes the weight and the vertical coordinate denotes the average connectivity.

**C.** WGCNA module clustering plot. The vertical axis indicates the coefficient of dissimilarity.

Abbreviations: WGCNA, weighted gene co-expression network analysis

**Figure S3 Validation of the diagnostic scoring model**

**A.** ROC curve of the diagnostic model in the training set.

**B.** Distribution of RiskScore in control and PCOS groups.

**C.** Heat map of the expression of the NDDGs in each sample.

Abbreviations: ROC, receiver operating characteristic curve; NDDGs, differentially diagnostic genes for necroptosis

**Figure S4 Validation of NDDGs in GSE84958**

**A-D.** The express level of TNFSF10, BCL2, PYGM and IL33 in GSE84958

(** p < 0.01)

**Supplementary Figures**

**Figure S1**


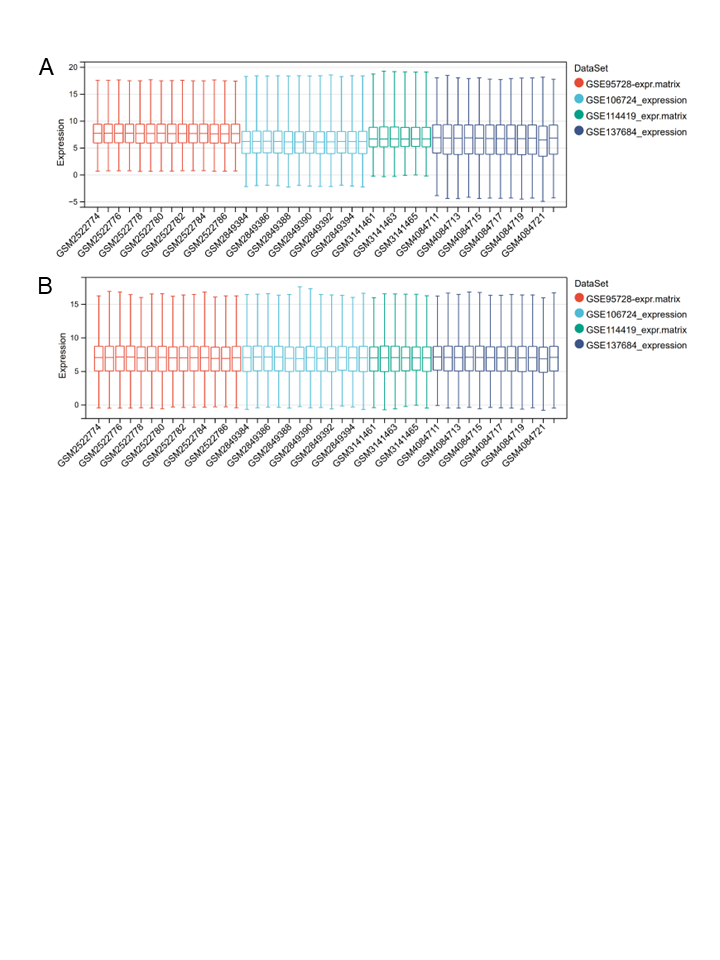


**Figure S2**


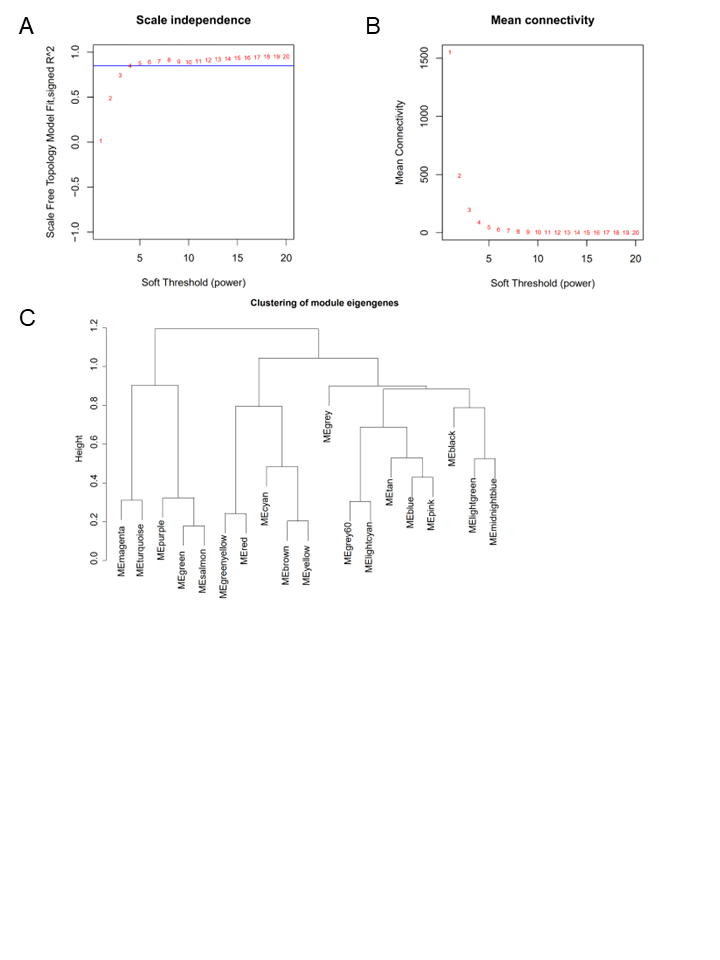


**Figure S3**


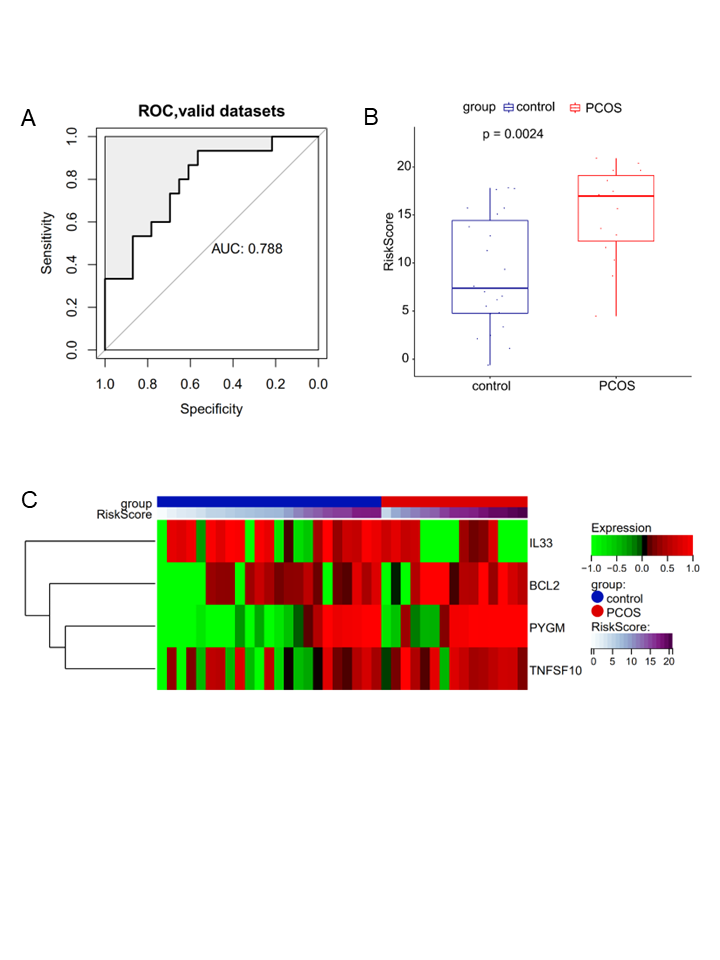


**Figure S4**


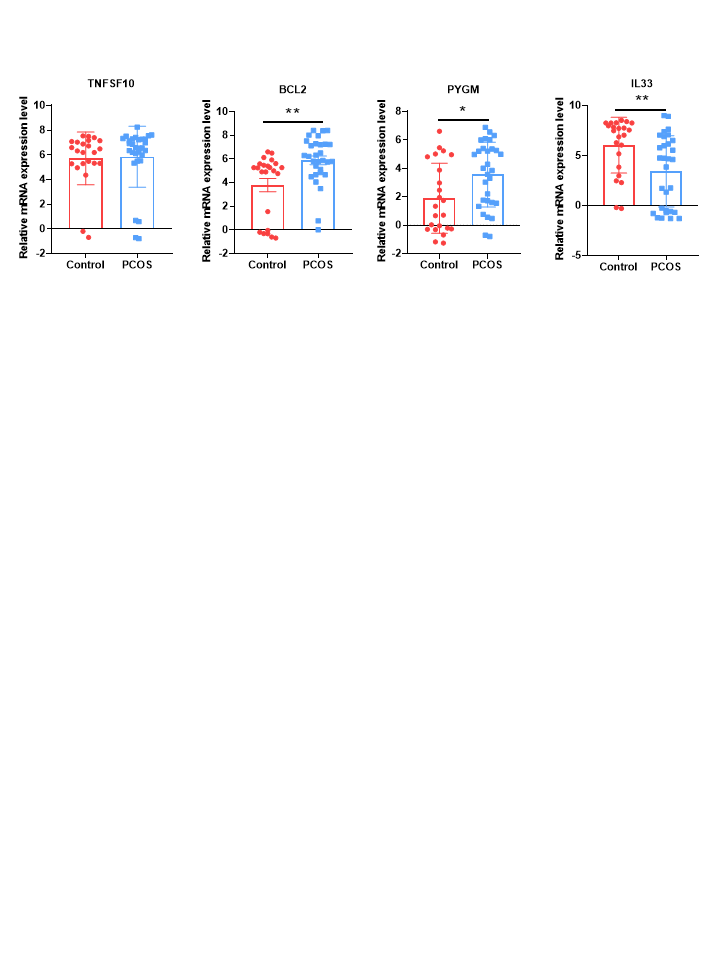

Supplement: Supplementary file 1 [file DataSheet_1.docx]
