# Supplementary material for: A novel model based on necroptosis to assess progression for polycystic ovary syndrome and identification of potential therapeutic drugs
Source: Front Endocrinol (Lausanne). 2023 Sep 7;14:1193992. doi: 10.3389/fendo.2023.1193992 (PMC10517861; doi:10.3389/fendo.2023.1193992)
Supplement: Supplementary file 2 [file DataSheet_2.docx]

Table S1 The primer sequences used were listed

| Genes | Primer sequence | | | |
| --- | --- | --- | --- | --- |
|  | mouse | | human | |
|  | Forward primer | Reverse primer | Forward primer | Reverse primer |
| TNFSF10 | ATGGTGATTTGCATAGTGCTCC | GCAAGCAGGGTCTGTTCAAGA | TGCGTGCTGATCGTGATCTTC | GCTCGTTGGTAAAGTACACGTA |
| BCL2 | ATGCCTTTGTGGAACTATATGGC | GGTATGCACCCAGAGTGATGC | \| GGTGGGGTCATGTGTGTGG \| \| --- \| | \| CGGTTCAGGACTCAGTCATCC \| \| --- \| |
| PYGM | CTTAGCCGGAGTGGAAAATGT | GTAATCTCTCGGAGTAGCCACA | CAATGTCGGTGGCTACATCCA | \| TATCTGGGAAGGCATCGAAGT \| \| --- \| |
| IL33 | TCCAACTCCAAGATTTCCCCG | CATGCAGTAGACATGGCAGAA | GTGACGGTGTTGATGGTAAGAT | AGCTCCACAGAGTGTTCCTTG |
